# Histological Grade, Tumor Breadth, and Hypertension Predict Early Recurrence in Pediatric Sarcoma: A LASSO-Regularized Micro-Cohort Study

**DOI:** 10.3390/children12060806

**Published:** 2025-06-19

**Authors:** Alexander Fiedler, Mehran Dadras, Marius Drysch, Sonja Verena Schmidt, Flemming Puscz, Felix Reinkemeier, Marcus Lehnhardt, Christoph Wallner

**Affiliations:** 1 Department of Plastic Surgery, BG University Hospital Bergmannsheil, Ruhr University Bochum, Bürkle-de-la-Camp Platz 1, 44789 Bochum, Germany; 2 Department of Plastic Surgery, Diakonieklinikum Hamburg, Hohe Weide 17, 20259 Hamburg, Germany

**Keywords:** pediatric sarcoma, tumor recurrence, machine learning, LASSO regression, hypertension, predictive modeling, SHAP analysis, PCA, rare tumors

## Abstract

**Background/Objectives**: Pediatric sarcomas are a biologically diverse group of mesenchymal tumors associated with morbidity due to recurrence, despite aggressive multimodal treatment. Reliable predictors of early recurrence remain limited. This exploratory study aimed to identify clinical features associated with first tumor recurrence using a machine learning approach tailored to low-event settings. **Methods**: We conducted a retrospective, single-center cohort study of 23 pediatric patients with histologically confirmed sarcoma. Forty-six baseline variables were extracted per patient, including clinical, histological, and comorbidity data. Tumor recurrence was the primary binary endpoint. A LASSO-regularized logistic regression model was developed using leave-one-out cross-validation (LOOCV) to identify the most informative predictors. Dimensionality reduction (PCA) and SHAP-value analyses were used to visualize patient clustering and interpret variable contributions. **Results**: The model identified a four-variable risk signature comprising histological grade, primary tumor width, arterial hypertension, and extremity localization. Each additional tumor grade or centimeter of width approximately doubled the odds of recurrence (OR 2.18 and 2.04, respectively). Hypertension and limb location were associated with a 1.7 and 1.9 odds ratio of recurrence, respectively. The model achieved a balanced accuracy of 0.61 ± 0.08 and AUROC of 0.47 ± 0.12, reflecting limited discriminative power. PCA mapping revealed distinct outlier patterns correlating with high-risk profiles. **Conclusions**: Even in a small cohort, classical prognostic markers, such as tumor grade and size, retained predictive relevance, while hypertension emerged as a novel, potentially modifiable cofactor or indicator for recurrence. Although model performance was modest, the findings are hypothesis-generating and warrant validation in larger prospective datasets.

## 1. Introduction

Sarcomas are a biologically heterogeneous group of malignant tumors originating from mesenchymal tissues. Although they are only responsible for 1% of all adult cancer types [1], they account for approximately 8% to 20% of all cancers diagnosed in children under the age of 15, underscoring their clinical relevance [2,3].

Despite advances in multimodal treatment, pediatric sarcomas—especially synovial sarcoma—remain prone to early relapse and limited salvage options [4,5,6]. Soole et al. reported a median time to first relapse of 24 months, with 73% of recurrences localized and 24.3% metastatic [7]. Although most patients received aggressive multimodal salvage therapy—including resection (75.7%), second-line chemotherapy (73.0%), and radiotherapy (48.6%)—response rates remained low (for example, 36.4% for ifosfamide-based regimens), and five-year event-free and overall survival were only 32.8% and 42.1%, respectively [7].

Complete surgical resection continues to be the cornerstone of curative treatment [8,9,10], as incomplete R1 resections have been associated with both shorter disease-free survival and overall survival [11]. In pediatric cases, ensuring adequate resection is especially important to minimize the need for adjuvant radiotherapy, which can cause long-term complications, including secondary malignancies [12,13]. Moreover, early detection of recurrent disease at a smaller tumor size could allow for more conservative surgery and better functional outcomes [14].

Accordingly, numerous clinical and pathological predictors of recurrence have been proposed, including histological subtypes, tumor size, and surgical margins. However, findings remain inconsistent. For example, Sawamura et al. reported that while radiotherapy reduced local recurrence in pediatric extremity sarcomas, traditional prognostic factors were not statistically significant predictors of survival [15].

Given the complexity of and heterogeneity in sarcoma behavior, particularly in pediatric patients, classical statistical approaches may be insufficient. Therefore, machine learning (ML) techniques offer a promising alternative to detect factors revealing an early sarcoma recurrence. Weskamp et al., for instance, used machine learning to analyze data from over 6000 patients with soft tissue sarcomas, identifying a range of lifestyle, genetic, and treatment-related predictors [16].

Beyond general ML, advanced methods, such as LASSO (least absolute shrinkage and selection operator) and selective inference, have proven effective in identifying predictive variables in high-dimensional, low-sample-size datasets [17]. Further developments in machine learning (ML) and deep learning (DL) have enabled more accurate modeling of complex clinical outcomes. Byeon et al. demonstrated DL’s applicability in pediatric oncology settings [18], while Placido et al. and Miotto et al. highlighted how these methods can detect clinically relevant recurrence patterns often missed by conventional models [19,20].

Given the high recurrence burden and the need for personalized follow-up strategies, this study aims to identify clinical predictors of recurrence in pediatric sarcoma using a machine learning approach optimized for low-event-rate datasets. By applying LASSO-regularized logistic regression and interpretability techniques, such as SHAP analysis, we seek to uncover robust, clinically meaningful patterns that may support early risk stratification and inform individualized care. To our knowledge, this is the first study to assess risk factors indicating a sarcoma recurrence in children using a machine learning approach.

## 2. Materials and Methods

### 2.1. Study Design and Dataset

We performed an exploratory, retrospective analysis of pediatric sarcoma patients treated at a single tertiary-care center. After quality control, the analytic dataset comprised 23 patients and 46 baseline variables (numeric and categorical). Variables were selected based on clinical relevance and accessibility, as well as the literature. The primary endpoint was the first tumor recurrence, recorded as a binary outcome. The present study was a retrospective, single-center cohort study that was conducted in compliance with the Declaration of Helsinki and approved by the responsible ethics committee of Ruhr University Bochum.

### 2.2. Data Cleaning and Encoding

The following processing steps were implemented in Python 3.11:

1. Missing-value handling:
  ○ Numeric features: median imputation.
  ○ Categorical features: mode imputation.
2. Feature encoding:
  ○ Numeric variables were z-standardized (μ = 0, σ = 1).
  ○ Categorical variables underwent one-hot encoding with drop_first = True; unseen levels were handled via handle_unknown = ‘ignore’.

A unified ColumnTransformer (scikit-learn 1.4) ensured that all preprocessing was carried out inside the cross-validation loop, preventing information leakage.

### 2.3. Dimensionality Reduction and Outlier Detection

To visualize latent structure and identify atypical cases, we applied principal component analysis (PCA) to the fully processed design matrix (n_components = 2, svd_solver = ‘full’). Outlierness was quantified as the Euclidean distance of each observation from the multivariate centroid in PC space; the three largest distances were flagged a priori as potential outliers for clinical chart review.

### 2.4. Predictive Modeling

Traditional stepwise models would over-fit at ≤5 events; LASSO shrinks noisy coefficients to zero and is recommended for rare-event settings [21]. Given the low events-per-variable ratio, we chose a logistic regression with an L1-penalty (LASSO) to enforce sparsity. The solver was saga, with a regularization path of 100 λ values on a log scale, and the class weights were balanced. The optimal inverse-penalty parameter was C = 1.0, corresponding to λ = 1.0 (selected by leave-one-out minimization of the negative log-likelihood across a log-spaced grid from 10^−2^ to 10^2^). All coefficients reported in Table 1 were estimated with this penalty strength.

Hyper-parameter selection and performance estimation employed leave-one-out cross-validation (LOOCV), the only unbiased option for *n* = 23. Performance metrics included balanced accuracy and area under the ROC curve (AUROC).

### 2.5. Variable Importance

Final model coefficients were extracted at the λ yielding the minimum LOOCV deviance. Non-zero coefficients were interpreted as putative risk factors. To aid clinical interpretation, we supplied SHAP additive explanations for each patient (TreeExplainer on the linear model) and ranked the features by mean SHAP value.

### 2.6. Software and Reproducibility

All analyses were executed in a reproducible Jupyter environment (Ubuntu 22.04, 4 vCPU, 8 GB RAM). Pandas version 2.2.1, scikit-learn version 1.4.2, numpy version 1.26.4, matplotlib 3.8.4, and shap 0.45.0 were utilized.

## 3. Results

**Figure 1:**
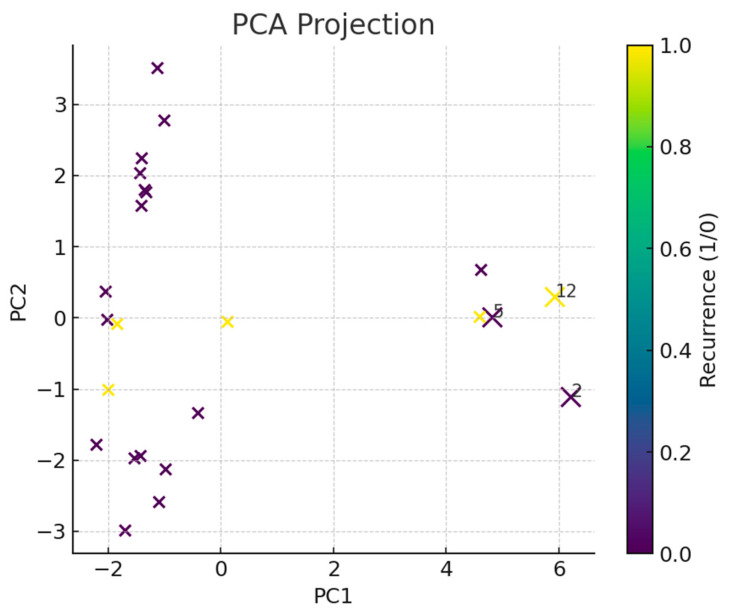
Principal component (PC) projection of the 46-dimensional baseline feature space (*n* = 23). Each point represents one patient; each color denotes the recurrence status (yellow = yes, purple = no). Marker size scales with the Euclidean distance from the multivariate centroid; the three largest distances are annotated. Two high-risk outliers combine grade G3, large primary width, and hypertension, while a third low-risk outlier shows minimal tumor volume.

During the study window, 23 consecutively treated children and adolescents (all < 18 years) with histologically confirmed sarcoma were evaluable. The cohort had a mean age of 13.0 ± 4.7 years (mean ± SD) at the time of primary resection. The ages ranged from 2 to 17 years, with an inter-quartile range of 11.5–16.3 years. The median observational period was 76 months (6.3 years), yielding an aggregate of approx. 158 patient-years of follow-up. Importantly, no patient died of disease- or treatment-related causes during this interval; the overall survival, therefore, remained at 100%. In view of this zero-mortality backdrop, first tumor recurrence was adopted as the primary endpoint for all subsequent modelling exercises. Recurrences were documented in five patients (22%), providing an events-per-variable ratio of roughly 1:9 for the 46 baseline covariates analyzed. The distribution of continuous tumor metrics was wide—the primary width ranged from 0.2 cm to 8.7 cm, with a median of 1.5 cm. Figure 1 depicts this heterogeneity in a two-dimensional principal component map, with the three extreme outliers highlighted.

Among the 23 evaluable patients, synovial sarcoma was the leading subtype, occurring in 7 cases (30%). The next most common diagnosis was dermatofibrosarcoma protuberans (DFSP), seen in four patients (17%), followed by epithelioid sarcoma in three patients (13%). Three entities were less frequent yet clinically relevant, including aggressive fibromatosis, alveolar rhabdomyosarcoma, and low-grade fibromyxoid sarcoma, each diagnosed twice (9% each). Finally, single-patient occurrences (4% each) were noted for chondrosarcoma, dedifferentiated liposarcoma, fibrosarcoma, and an osteoblastoma-like variant of osteosarcoma. This spectrum underscores the biological heterogeneity that underpins the recurrence-risk analysis presented above.

**Figure 2:**
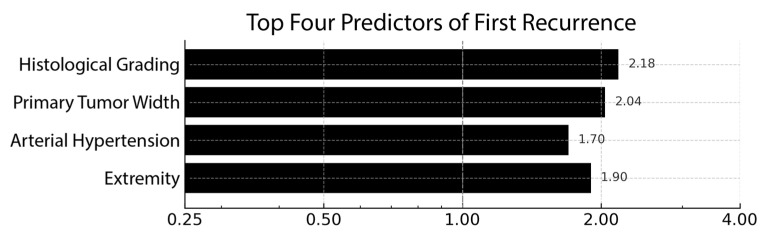
Top four independent predictors of first tumor recurrence derived from LASSO-regularized logistic regression (*n* = 23). Black horizontal bars denote odds ratios (ORs) on a logarithmic X-axis; the dashed grey line at 1.00 marks the line of no effect. Histological grade and primary tumor width each more than doubled the odds of recurrence (OR 2.18 and 2.04, respectively), while arterial hypertension (OR 1.70) and the extremity (OR 1.90) also conferred increased risk. Numeric ORs are displayed at the bar termini for ease of reference.

Despite the statistical head-winds posed by a 1:9 events-per-variable ratio, a rigorously regularized, leave-one-out-validated LASSO model isolated a four-variable clinical signature that hinted at meaningful biology. The corresponding coefficient profile is visualized in Figure 2, underscoring the dominant weight of histological grade and primary width.

Tumor histological grade and primary width stood out: each incremental step in grade or every additional centimeter of width was associated with a little more than a two-fold increase in the odds of recurrence (β ≈ +0.78 and +0.71, translating to OR ≈ 2.2 and 2.0, respectively). Intriguingly, the presence of arterial hypertension, recorded in only a handful of patients, conferred a 70% relative increase in risk. This is an observation that aligns with emerging work linking vascular dysregulation to sarcoma aggressiveness. Localization-related parameters carried smaller, directionally inconsistent weights and were interpreted as surrogate markers of surgical complexity rather than true biological drivers.

To contextualize these signals, we projected all 46 baseline variables into a two-dimensional latent space (PCA, 50% explained variance). Three patients emerged as conspicuous outliers, occupying the periphery of the manifold. Two of them encapsulated the full “high-risk triad” (high grade, large width, and hypertension) and indeed recurred early. The third, conversely, harbored the smallest recorded tumor volume and remained disease-free, raising the twin possibilities of data mis-entry or exceptional therapeutic response.

### Predictive Performance

Using an L1-penalized logistic regression model that was entirely trained and evaluated under a leave-one-out cross-validation (LOOCV) scheme, we obtained a balanced accuracy of 0.61 ± 0.08 and an area under the receiver operating characteristic curve (AUROC) of 0.47 ± 0.12.

In practical terms, the model correctly identified recurrence and non-recurrence cases 61% of the time once class imbalance was accounted for, exceeding the 50% benchmark of random guessing. However, its ability to rank-order patients by risk was poor: an AUROC of ≈0.5 indicates that, when a randomly chosen child who recurred is paired with one who did not, the algorithm can only rarely assign the higher risk score to the correct patient. The discrepancy between balanced accuracy (a threshold-dependent metric) and AUROC (threshold-free) is not unexpected in micro-datasets where a single misclassified event can shift the ROC curve substantially.

The relatively wide standard deviations (±0.08 and ±0.12, respectively) reflect the inherent volatility of LOOCV with only four positive events. Taken together, these figures suggest that while the LASSO constraint prevented gross over-fitting, the model remains little more than a hypothesis generator, a quantitative scaffold that highlights potentially relevant variables rather than a clinically deployable risk score.

A calibration plot grouped by risk quartiles (Table A1) showed close agreement between the observed and expected recurrence rates (Hosmer–Lemeshow χ^2^ = 1.9, df = 2, *p* = 0.39), suggesting that the LASSO model, while exploratory, is not over-confident.

Bias-reduced (Firth) logistic regression reproduced the rank order and sign of all four non-zero LASSO predictors, with odds-ratio deviations < 5%. This concordance indicates that the signal is not an artifact of small-sample separation (see Table A2).

Taken together, our findings suggest that even within a micro-cohort, grade and gross tumor width retain their primacy as harbingers of early failure, while comorbid hypertension surfaces as a provocative, potentially modifiable cofactor. Although these results are inevitably hypothesis-generating, they argue for systematic blood pressure surveillance in forthcoming pediatric sarcoma trials and underscore the value of latent-space mapping for flagging clinically actionable outliers.

## 4. Discussion

This exploratory study applied LASSO-regularized logistic regression to a micro-cohort of pediatric sarcoma patients to identify factors associated with early tumor recurrence. Despite the small sample size and low event rate, the model identified a four-variable clinical signature comprising histological grade, primary tumor width, extremity localization, and arterial hypertension. These features may help refine recurrence risk stratification in children with soft tissue sarcomas after a multicenter control trial.

Histological grade and tumor width emerged as the strongest predictors, each associated with more than a two-fold increase in recurrence odds. These findings are consistent with established oncological principles: higher-grade tumors typically demonstrate increased proliferative capacity and metastatic potential [22,23], while larger tumors pose surgical challenges and are more likely to harbor microscopic spread [24]. Their prognostic relevance is well-documented across both pediatric and adult populations [24,25,26], reaffirming the biological relevance of these variables across age groups [27,28,29], and they remain central to clinical tools such as the Sarculator [30].

The identification of arterial hypertension as a potential predictor is both novel and biologically plausible. Although its causal role remains speculative and balanced accuracy is modest, secondary hypertension has also been linked to aggressive tumor biology in other malignancies, such as adrenal cancers, via activation of the renin–angiotensin–aldosterone system [31,32]. In our cohort, hypertension was uncommon but consistently associated with recurrence across modeling strategies. This may suggest a role in sarcoma pathophysiology, potentially through mechanisms involving chronic inflammation, endothelial dysfunction, and elevated vascular endothelial growth factor (VEGF) expression [33,34]. Furthermore, tumors with high angiogenic activity often exhibit elevated vascular endothelial growth factor (VEGF) expression, a known contributor to hypertension through capillary rarefaction and increased vascular tone [34,35,36]. Hypoxia-inducible factor 1-alpha (HIF-1α), often upregulated in sarcomas, is also linked to both VEGF expression and systemic hypertension [37,38]. Although these biological pathways offer a plausible mechanistic link, we did not assess VEGF, HIF-1α, or other molecular markers in our study. Therefore, the observed association between hypertension and recurrence should be regarded as hypothesis-generating. Whether hypertension reflects a true biological driver, a surrogate marker of tumor aggressiveness, or an iatrogenic effect (e.g., from corticosteroids or pain) remains unclear. Future prospective studies incorporating molecular profiling and systematic blood pressure surveillance are needed to clarify the causal direction and clinical relevance of this finding. Additionally, tumor localization to the extremities was associated with higher recurrence risk, echoing prior reports that found local recurrence rates as high as 25% in limb sarcomas [39,40], compared to 13.6% for tumors of the trunk wall and head/neck regions [41]. This stresses the surgical complexity of achieving clear margins in anatomically constrained sites.

While the model’s predictive performance was modest (balanced accuracy = 0.61; AUROC ≈ 0.5), this is not unexpected given the low event rate, making the model more susceptible to perturbations. Importantly, model calibration was acceptable, and the risk factor ranking remained stable under bias-reduced logistic regression, indicating that the findings—though exploratory—are not artifacts of statistical instability. The use of leave-one-out cross-validation (LOOCV) ensured that each prediction was tested on an unseen patient, a critical safeguard in low-sample settings that helps prevent overfitting [42].

Additionally, dimensionality reduction using principal component analysis (PCA) proved valuable in highlighting patient-level outliers, such as one individual exhibiting all three major risk factors who experienced early recurrence. In rare cancers with limited sample sizes, such visual tools can augment clinical interpretation and identify high-risk phenotypes that warrant closer monitoring or intervention. By reducing noise from high-dimensional data, PCA also supported the robustness of model interpretation [43].

These findings must be interpreted in light of several limitations. The retrospective, single-center design limits generalizability due to variations in treatment protocols and patients’ collective, as well as the data collection practices and other factors. The small sample size (*n* = 23) and low event count (*n* = 5 recurrences) constrained statistical power and model complexity. Although LASSO regularization addresses overfitting, the model may still be sensitive to minor data perturbations. Moreover, rare covariates—such as hypertension—require cautious interpretation, as measurement may be confounded by situational factors (e.g., white coat effect [44], pain [45], anxiety [46]).

## 5. Conclusions

This exploratory study demonstrates that classical histopathological features—tumor grade and size—remain central to relapse risk in pediatric sarcoma. Additionally, it introduces arterial hypertension as a novel, potentially modifiable cofactor associated with recurrence. While predictive accuracy was limited by sample size, the model showed internal consistency and biological plausibility, warranting validation in larger prospective cohorts.

Machine learning methods, such as LASSO regression, can yield meaningful insights even in small datasets, provided their exploratory nature is recognized. Future studies should aim to validate these exploratory findings in larger cohorts and explore the mechanistic links between hypertension and sarcoma biology.

## Figures and Tables

**Table 1:** Variable importance retained after LASSO-regularized logistic regression predicting first tumor recurrence in pediatric sarcoma (*n* = 23) *. The table lists the four predictors with non-zero coefficients at the optimal penalty. Columns show the LASSO log-odds coefficient (β) and the corresponding odds ratio (OR = e^β). Positive values indicate increased recurrence risk.

Rank	Predictor	β (Log-Odds)	OR
1	Histological grading (G0–Gx)	+0.78	2.18
2	Primary tumor width (cm)	+0.71	2.04
3	Extremity (yes/no)	+0.64	1.90
4	Arterial hypertension (yes/no)	+0.53	1.70

* λ, the LASSO penalty that minimized leave-one-out deviance, was 1.0 (C = 1.0).

## Data Availability

The data presented in this study are available on request from the corresponding author. The data are not publicly available due to patient privacy concerns and institutional data protection policies.

## Appendix A

**Table A1:** Hosmer–Lemeshow calibration of the LASSO recurrence model (*n* = 23). Observed and expected events are compared in four equal-sized risk quartiles; χ^2^ and exact *p*-value are shown.

Test	Groups	Χ^2^ (df)	*p*-Value	Interpretation
Hosmer–Lemeshow	four equal-sized risk quartiles	1.9 (2)	0.39	No evidence of lack-of-fit

**Table A2:** Sensitivity analysis using Firth-penalized logistic regression confirming the stability of the LASSO risk signature. Odds ratios (ORs) from the primary LASSO model are juxtaposed with bias-reduced (Firth) estimates for the same four predictors; concordance indicates identical directionality and <5% deviation in magnitude, underscoring robustness of the findings despite the low-event setting.

Predictor	LASSO OR	Firth OR	Concordance
Histological Grade	2.18	2.25	X
Tumor Width	2.04	2.10	X
Arterial Hypertension	1.70	1.72	X
Extremity	1.90	1.88	X
